# An Ethogram of the Reproductive Behaviour of the American Mink (*Neovison vison*) in Farmed Conditions

**DOI:** 10.3390/ani13030443

**Published:** 2023-01-28

**Authors:** Beata Seremak, Aleksandra Wojciechowska, Bogumiła Pilarczyk, Agnieszka Tomza-Marciniak

**Affiliations:** Department of Animal Reproduction Biotechnology and Environmental Hygiene, Faculty of Biotechnology and Animal Husbandry, West Pomeranian University of Technology, Janickiego 29, 71-270 Szczecin, Poland

**Keywords:** American mink, *Neovison vison*, reproduction, reproductive behaviour, ethogram

## Abstract

**Simple Summary:**

Behavioural analysis can be one of the elements that allows an assessment of the conditions in which animals are kept and their welfare. Animals kept in farmed conditions should be able to freely express their typical behaviour. The aim of the present study was to create an ethogram of the reproductive behaviour of American mink kept in farmed conditions. Reproductive behaviours include: chasing the female, grasping her neck with the teeth, marking the local area, mounting the partner, and sniffing the neck, anal and genital areas. The obtained results indicate that the American mink can express some natural reproductive behaviour in breeding conditions, which may also be an indicator of the welfare of these animals.

**Abstract:**

Ethograms can serve as benchmarks against which abnormal animal behaviour can be identified, and then normal behaviour can be restored by appropriately modifying the environment in which an individual resides. The aim of the present study was to create an ethogram of the reproductive behaviour of American mink kept in farmed conditions. The research material consisted of 12 one-year-old male American mink, pearl coloured, selected randomly from among the varieties of coloured mink on the farm, and 60 two-year-old females. The animals participating in the study were grouped into two breeding sets, each consisting of 30 females and 6 males. Reproductive rituals included chasing the female, grasping her neck with the teeth, marking the local area, mounting the partner, and sniffing the neck, anal and genital areas. The copulation times recorded in this study varied greatly, ranging from several minutes to over two hours. The obtained results indicate that the American mink can express some natural reproductive behaviour in breeding conditions, which may also be an indicator of the welfare of these animals.

## 1. Introduction

In recent years, the breeding of fur animals has become the subject of lively discussion in the public sphere, with public opinion being generally interested in their welfare and living conditions. Currently, the improvement of animal welfare, especially of those used in cage farming, is an important task for the European Union. A key element in assessing animal welfare is behavioural analysis, which can provide an insight into the conditions under which the animals are kept. Indeed, previous attempts to enrich the breeding environment of mink by introducing additional cage equipment have had a positive impact on their welfare [1].

Animals kept in farmed conditions should be able to freely express their typical behaviour, and the appearance of abnormal behaviour is often the first sign of problems.

Banks [2] defines animal behaviour as a series of motor activities, vocalisations and odours related to bodily functions and social interactions. Behaviour develops from environmental and genetic factors as a dynamic process that is particularly sensitive to changing physical and social factors [3].

One of the most important tools in the study of animal behaviour is the ethogram, which Banks [2] defines as a detailed catalogue of behaviours typical of a given species. A given behaviour is believed to arise in response to a set of external and internal stimuli, as well as the processes taking place in the animal itself. Behaviours are an uninterrupted sequence of identifiable movements and events, whose form, speed, duration, strength and orientation can be described in detail to create a behavioural pattern [4].

These are divided into two main categories: individual behaviour and social behaviour [5]. Behavioural patterns form complex behavioural systems, which Pellis et al. [6], Baerends [7] or Tinbergen [8] define as structurally or functionally coherent sets of related cause-effect and hierarchically-coordinated behavioural patterns, whose expression, organisation and coordination can be controlled by highly-specific environmental stimuli, sensory-motor processes and motivational mechanisms. In other words, they can be regarded as a group of behavioural patterns that share a common general function and, typically, a common basis. Behavioural systems are quite similar to physiological systems and demonstrate direct relationships with each other, such as between sexual behaviour and the reproductive system. In addition, like physiological systems, behavioural systems do not function completely independently of each other [9,10].

Behaviour is commonly researched using ethograms. An ethogram is a record that describes all types of behaviour encountered during behavioural studies [11,12]. It should use a classification method that allows behaviours to be matched unambiguously to single, distinct categories [13]. The frequency and duration of each given behaviour should be estimated using an appropriately selected observation method [14]. The choice of method is influenced by various factors, such as the time that can be devoted to the observation, the size of the observed group of animals, the number of people involved and the budget [13,14,15]. In many studies, the researchers develop only partial ethograms, e.g., those relating only to specific behaviours relating to the subject of the study [16].

From the point of view of reproductive processes, the American mink is a very interesting species. It is characterised by a monoestral type of reproduction, the occurrence of delayed implantation of blastocysts and a varied gestation length [17,18]. They reach sexual maturity at the age of 8–12 months, manifested by the appearance of permanent morphological and hormonal changes in the reproductive system; these consist of folliculogenesis, ovulation, luteinisation and luteolysis of the corpus luteum, i.e., the appearance of a permanent oestrous cycle. In the northern hemisphere, the reproductive season of the American mink is relatively short, with oestrus being stimulated by an increase in day length [19]. The reproductive period is estimated to last from two to three weeks [20,21] to one month [22]. In farm conditions, the cyclical maturation of ova during the oestrus period makes it possible for mink to be mated multiple times with the same or different males [21]. In female mink, copulation, ovulation and fertilisation do not shorten the oestrus period [23], and blastocysts from the first mating continue to develop in spite of subsequent mating attempts [22,24,25].

Various mating schemes are employed in mink farming. These can be double, according to the formula 1 + 8 or 1 + 9 (the numbers indicate the days in the reproductive cycle on which mating occurs), triple, according to the formula 1 + 2 + 8, 1 + 2 + 9 or 1 + 8 + 9, and quadruple ones, according to the formula 1 + 2 + 8 + 9. However, some females are mated only once during the entire reproductive period.

In its natural environment, the mink is highly territorial, and marks the borders of its own territory with secretions from its anal glands. In the wild, male mink migrate to female territories during the breeding season, however, in farm rearing, the females are transferred to the male territory, i.e., the cage. The optimised mating conditions present on farms can generate stress for both males and females, especially those that are not ready or willing to mate. This is manifested by the occurrence of certain types of behaviour.

The aim of the present study was to create an ethogram of the reproductive behaviour of American mink kept in farmed conditions.

## 2. Materials and Methods

The experiment was carried out in breeding seasons on a mink farm located in the Zachodniopomorskie (West Pomeranian) Voivodeship, in Poland. All mink were kept in accordance with the European Convention for the Protection of Vertebrate Animals and met the conditions of the Act of 29 June 2007 in force in Poland, and the Minister of Agriculture and Rural Development Regulation of 10 September 2015 on the minimum conditions for keeping farm animal species, valid since 1 January 2018. In accordance with Polish law, due to the non-invasive nature of the procedures, the present study did not require the consent of the ethical committee for animal research [26].

The animals included in the study did not show any behavioural disturbances. The animals were managed in an open-shed system and fed a standard semiliquid feed based on chicken and fish (protein: 45–50%, fat: 35–40%, carbohydrates: 12–18%) and mineral-vitamin supplements. The mink were housed individually in standard cages (L/W/H = 90/45/45 cm) without any additional environmental equipment. The cages consist of an enclosure for the animals and a litter house with a mesh insert at the front. The cages are located approximately 70 cm above the ground and are equipped with automatic nipple drinkers, providing the animals with constant access to clean and fresh water. The feed was distributed using semi-automatic feeding machines with combustion engines and provided directly to the cages using an automatic dispenser twice a day, i.e., at 5.30 and 16.00.

The research material consisted of 12 one-year-old male American mink, pearl coloured, selected randomly from among the varieties of coloured mink on the farm, and 60 two-year-old females. The animals participating in the study were grouped into two sets, each consisting of 30 females and 6 males. Such breeding sets are commonly used to optimise mating procedures.

The females were mated according to the following scheme: 1 + 2 + 8 + 9, i.e., mating on the first, second, eighth and ninth days of sexual activity. During the breeding period, the mink were typically mated within one set; however, where males showed no desire to mate, mating with females from another breeding set was allowed.

Each given day, the females were presented to the males twice: from 7.00 and from 12.00. In the case of successful mating in the morning, another female was presented at noon. If the 07.00 mating or 12.00 mating was unsuccessful, the female was exchanged for another. This allowed the males to potentially cover two females a day.

Data were collected by serial recording. This approach allowed the collection of all behaviours of male and female mink tested during the reproductive period, which in this case lasted from 6 to 17 March. Observations were performed every day from 6.00 a.m. to 6.00 p.m. A total of 1728 h of recorded footage were analysed. The obtained data provided an insight into the frequency, duration, sequence of occurrence of specific behaviours and interactions between individuals.

Video-recorded material of the male and female mink in the peri-copulatory period was analysed using the Behawior program.

Mink behaviour was analysed in relation to the seven most common categories of reproduction behaviour, as proposed by Scott [5]:

Rest—the male shows no interest in his surroundings, takes a relaxed posture or sleeps with the body curled up.
Grooming—the male cleans the genital area and fur with his mouth or paws.
Observation—the male rests his front paws on the chest, stands erect and looks around, or lies with his eyes open, listening and carefully observing his surroundings.
Copulatory behaviour—the male sniffs the female and around her genitals, the male climbs the female, the animals remain motionless during copulation, roll over to the side, the male grabs the female by the neck with his teeth and chases her.
Play—the male rolls on his back, rubs against the cage and waves his paws.
Stays in the house—the male is outside the cage.
Other behaviours—for example freezing, defecating, eating, listening or occasional head banging.

## 3. Results

Appropriately marked behaviours characteristic for both sexes, or for males or females alone, were also classified in the study (Table 1).

The observed females reacted differently to the presence of males. Attempts to copulate and copulation itself were observed, as well as attempts to encourage the male to copulate by rubbing against him with the whole body. A lack of interest or aggressive behaviour towards the male (two cases) were also observed, as well as avoiding contact with the male. In some cases, the female prevented the male from grasping the neck with the teeth and attempted to break free from the grip during copulation. No sterile females were recorded in the experimental group.

Extreme behaviour was observed among males. Some (*n* = 3) showed little or no interest in females: in such cases the males explored the surroundings while the female was in the cage or hid in the spawning room and stayed there until the female was removed. In contrast, other males (*n* = 2) showed great interest in females, started copulation quickly and did not allow the female to free herself from their grip.

The males were noted to mark their cages with urine and faeces and rub their belly or sides against the cage. Copulation was initiated by grasping the female’s neck with the teeth.

A total of 213 copulations lasting more than 10 min were analysed and 14 were classified as samples (30 s to 6 min). The copulation times recorded in this study varied greatly, ranging from several minutes to over two hours (Table 2).

Among the twelve males, the highest number of successful copulations during the breeding season was observed in male number 10 (26 attempts), followed by male number 4 (24 attempts). These males were characterised by a strong temperament, impetuous behaviour and showed greater interest in females than the other males. These animals were more eager to copulate, and also prevented the female from escaping before or during copulation by pinning her to the ground and gripping her nape or tail firmly with their teeth. In the event that the female escaped, the males immediately gave chase.

The lowest number of matings was recorded in males numbered 5, 3 and 1. These took part in 12, 10 and 7 coverings, respectively, during the breeding season. These males showed little or no interest in females. While the female was in the cage, these males would sniff the surroundings or retreat to the nesting box and remain there until the female was removed. During the day, the same males also demonstrated different behaviours towards individual females.

The shortest copulation time (qualified as effective) during the reproductive season, amounted to 13 min and 11 s. During the study, 14 copulations (lasting from 30 s to 6 min and 49 s) were recorded and classified as copulation attempts.

## 4. Discussion

As a given species may have an extremely diverse repertoire of behaviours, which may be difficult to assign to a specific category, an ethogram must employ certain points of reference [27,28]. Although the literature includes many observational reports of the copulatory behaviour of wild American mink, the species still lacks a typical ethogram. Therefore, the present analysis of recorded behaviours was based on the reproductive behaviour of other mustelids, including the European polecat and domestic ferret [29,30,31], black-footed polecat [32] and European mink [33].

**Figure 1:**
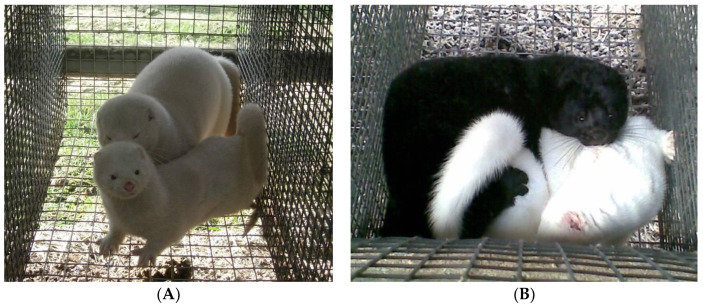
A female in a male’s cage (**A**), Trying to mount a female (**B**) (A. Wojciechowska).

According to Amstislavsky and Ternovskaya [34] sexual behaviour differs between mustelidae species. In the case of stoat (*Mustela erminea*), a male copulates with a young female in oestrus almost immediately after they are brought together, but sexually mature female European mink (*Mustela lutreola*) and sable (*Martes zibellina*), when in oestrus, may reject the breeding male, even if he is sexually mature and eager to copulate. In this study, several types of reaction to the presence of a partner were observed: commencing mating, attempting to mate, lack of interest, encouragement to mate by the female, and aggressive behaviour between individuals (recorded only twice) (Figure 1). Some of the observed reproductive behaviours of farmed mink are similar to those of free-living mink and other animal species. For example, in some cases, mink copulation was preceded by “courtship”, which resembled a struggle or a chase around the cage, with the male keeping a close distance behind the female. As noted by Dunstone [35], male wild American mink chase females in order to mate. Such “courtship”, in the form of a chase preceding mating, was also noted by Lodé [36] during a two-year observation of pairs of beech martens (*Martes foina)* caught from the wild and then kept in outdoor enclosures. Similar behaviour was also observed by Poole [29] in polecats (*Mustela putorius*) kept in an indoor experimental hall. However, in this species, the only examples of “chasing” were observed among males staying with females who were not in heat or unwilling to copulate. In our research, “chasing” was observed in males with both the highest and the lowest number of successful copulations; however, not all mating attempts were preceded by a chase.

During our observations, it was noticed that male mink marked the cages with urine and faeces and rubbed their belly or sides against the mesh. Similar behaviour has previously been noted in male free-living mink, which also marked their territory with urine, faeces and anal gland secretions [35]. Additionally, females use the scent to advertise sexual receptivity and fertility. This chemical signalling is observed in many species of mammals, including the ringtailed lemur, meadow vole, golden hamster or Canidae [37]

In addition, the males intensely sniffed their partner’s neck, anus and genital areas before copulation. Chemical compounds found in faeces, urine and glandular secretions serve as chemosensory clues, and play a special role in monoester animal species, including the American mink [38,39,40,41]. In turn, Berzins and Helder [42] tested the response to smells characteristic of the genitourinary system and the anal area in male and female ferrets (*Mustela putorius f. furo*); more specifically: the degree of interest in the smells, the ability to distinguish them and the use of this ability during the reproductive season. Both male and female ferrets were able to recognise known individuals based on scent and distinguish them from those they met for the first time. Brinck et al. [43] and Fleming [44] propose that males are able to determine whether a female has copulated before, and with which male, and identify the current phase of her reproductive cycle, by sniffing the perianal area and genitals. In addition, sniffing the back can detect bites and saliva residue that could indicate contact with another male. Similar pre-copulatory behaviours have been observed in common polecats [29], European mink [33] and beech martens [35].

Our findings indicate that the tested females reacted in different ways to the male and his attempts at copulation. Some took a defensive posture, trying to avoid contact with the male, making it difficult for him to grasp the neck with their teeth, or trying to free themselves from his grip during copulation. Others demonstrated an interest in the male and a willingness to mate. These behaved calmly and allowed the male to mount them. Interestingly, some females may have tried to encourage the male to copulate by rubbing against him with their whole body, despite him not being interested.

As previously emphasised, it is important to understand the species-specific behaviour of a species and its manifestation in the wild to ensure appropriate conditions in farmed breeding. Such animal welfare translates into improved health among kept animals, and thus greater economic performance. MacKinnon et al. [45] propose that poor reproductive performance may be caused by behavioural disturbances related to aggression or a lack of interest in a potential reproductive partner. This was confirmed by Kiik et al. [46], in a study carried out in the period 2004–2010 comparing the reproductive behaviour of wild European mink with those born in captivity and kept in Tallinn Zoo. During the study, 579 mating attempts were recorded, of which 147 (25%) were classified as successful and 432 (75%) as unsuccessful, mainly due to aggressive behaviour by the partner or her lack of interest. A relationship between male aggression and mating effectiveness was also noted by Andersen [47].

In American mink, copulation is initiated by the male grasping the neck of the female with his teeth ensuring a stable grip during copulation. In the case of mustelids, this grip is reinforced by the subcutaneous fat layer in the area. According to Enders [48], when the male is able to firmly grasp the neck with his teeth, mating usually occurs, irrespective of whether the partner wishes to do so. Such pre-copulatory behaviour was also noted by Kneidinger et al. [33] in European mink in captive breeding and by García [49] in American mink in their natural environment. Characteristic mustelid pre-mating behaviour was also reported by Wolf et al. [50] in the black-footed ferret. Kneidinger et al. [33] also observed in male European mink “clucking”, “flehmen”, “chase”, “anal drag”, and “mounting”.

In our present study, three males demonstrated extreme behaviour, i.e., either showing minimal interest in the female or completely ignoring her. In such cases, when the animals were together in the cage, the males examined the surroundings or hid in the nest box and stayed there until the female was removed from their cage. In contrast, two other males showed great interest in females, commencing mating quickly and not allowing them to free themselves from their grip throughout its duration.

The copulation times recorded in our present study varied greatly, ranging from several minutes to over two hours, which is consistent with the observations of Spangberg [51] or Malmkvist et al. [52]. The completion of mating, as described by Enders [48], was recognised by the straightened spine of the male, the curved tail of the female and involuntary movements of the hind legs. Similar to the beech marten [36], after a successful mating procedure, the animals showed no interest in each other and vigorously licked the genital area, abdomen and back.

In Europe, the American mink is considered an invasive species, which has negative effects on native ecosystems by disrupting population dynamics and causing a decline in populations of indigenous species [53]. The demonstration by farmed mink of reproductive behaviour, typical of free-living animals, creates the possibility of breeding these animals with free-living individuals, in the event of escape from the farm. This may pose a threat to biodiversity in a given area.

## 5. Conclusions

Our present findings were used to create an ethogram of reproductive behaviour of American mink kept in farmed conditions; this provides an insight into the behaviour patterns of the studied animals. Reproductive rituals included the male chasing the female, grasping her neck with the teeth, marking the local area, mounting her, and sniffing the neck, anal and genital areas. The obtained results indicate that the American mink can express some natural reproductive behaviour in breeding conditions, and that this may serve as an indicator of the welfare of these animals.

## Figures and Tables

**Table 1:** Ethogram of American mink reproductive behaviour in farmed conditions (♀—female, ♂—male).

Behaviour (♀/♂)	Definition	Frequency
Deliberately approaching partner (♀♂)	Approaching a partner.	385
Acceptance of male by female (♀)	The female tightens the skin at the nape of her neck or does not run away as the male climbs onto her.	312
Deliberately approaching markings (♀♂)	The animal walks towards marks left by the partner e.g., urine, faeces or scent.	307
Sniffing the genitals (♀♂)	The male/female sniffs the genital area of the partner.	305
Sniffing the neck/ fur (♀♂)	The male/female sniffs the area of the back of the neck or the partner’s fur.	287
Mounting (♂)	The male lays on the female, holding her hind legs in a position that allows the insertion of the penis.	213 + 14
Intromission (♂)	The moment of introducing the penis, characterised by an extreme arching of the male’s back.	213 + 14
End of copulation (♂)	The end of copulation is characterised by a straightening of the male’s back, the separation of partners and involuntary movements of the hind legs.	227
Copulation (♂)	Long-term (>10 min) mounting of the female by the male, (weak frictional/pelvic movements lasting from 3 s to 6 s were observed in some males)	213
Care (♀♂)	Males/females lick the genital area vigorously after copulation is complete.	163
Rubbing against the partner (♀♂)	Rubbing the whole body against the partner.	158
Finding a partner (♀♂)	The male or female walks around the cage, sniffing and exploring the surroundings.	149
Chasing (♂)	The animals chase each other in the cage prior to attempting to mate.	86
Biting the neck (♂)	The female is held or pulled by the male, who grips the skin on the back of her neck with his teeth.	85
Tail biting (♂)	The male grabs the female’s tail with his teeth, sometimes pulling her to him.	78
Marking the site (♂)	Marking the area with a scent by rubbing the sides of the body, the abdomen or anus against the cage, leaving urine or faeces on the mesh.	68
Lack of acceptance of the male by the female (♀)	The female moves away from the male attempting to mate or tries to free herself from him.	23
Attack/aggression (♀♂)	Agonistic behaviour designed to scare or hurt a partner.	2

**Table 2:** Duration and number of copulations of experimental males during the breeding season.

Male Number	Number of Coverings	Mean Copulation Time	SE	Min.	Max.
		[h:m:s]		[h:m:s]	[h:m:s]
1	7	01:06:46	00:09:04	00:24:00	02:15:20
2	20	01:01:07	00:05:11	00:21:20	01:42:53
3	10	00:59:33	00:08:47	00:17:50	01:34:24
4	24	00:31:37	00:02:53	00:13:46	00:59:19
5	12	01:11:14	00:10:39	00:19:43	02:28:13
6	22	00:55:36	00:10:02	00:14:24	02:04:42
7	22	00:58:27	00:06:55	00:20:15	02:07:05
8	15	00:48:30	00:07:03	00:14:37	01:55:08
9	18	01:06:15	00:05:28	00:40:14	01:57:38
10	26	00:48:46	00:04:19	00:13:11	01:36:13
11	21	01:14:17	00:05:20	00:24:48	02:01:25
12	16	01:14:23	00:07:12	00:25:30	02:17:41
Total	213				

## Data Availability

The data presented in this study are available on request from the corresponding author.
